# Lidocaine Combined With Platelet‐Rich Plasma for Stellate Ganglion Block in Chronic Migraine: A Multicenter, Prospective, Propensity Score‐Matched Cohort Study

**DOI:** 10.1002/brb3.71736

**Published:** 2026-08-31

**Authors:** Lu Liu, Baolin Duan, Tao Ma, Long Li, Dong Yang, Fang Luo

**Affiliations:** ^1^ Department of Day Surgery Center Beijing Tiantan Hospital Capital Medical University Beijing People's Republic of China; ^2^ Department Director of Pain Management Suzhou Blue Cross Brain Hospital Suzhou Jiangsu People's Republic of China; ^3^ Department of Pain Management Qinghai Provincial People's Hospital Xining Qinghai People's Republic of China; ^4^ Department of Pain Management Union Hospital, Tongji Medical College, Huazhong University of Science and Technology Wuhan People's Republic of China; ^5^ Key Laboratory of Anesthesiology and Resuscitation (Huazhong University of Science and Technology) Ministry of Education Wuhan People's Republic of China; ^6^ Department of Pain Management and Day Care Surgery Beijing Tiantan Hospital Capital Medical University Beijing China

**Keywords:** chronic migraine, monthly headache days, platelet rich plasma, stellate ganglion block

## Abstract

**Objectives:**

To explore the association of stellate ganglion block with platelet‐rich plasma plus lidocaine with clinical outcomes in adults with chronic migraine, in comparison to lidocaine alone.

**Methods:**

This multicenter prospective exploratory study enrolled 200 adults with chronic migraine scheduled for stellate ganglion block across three hospitals from October 2024 to September 2025. Treatment allocation was based on patient preference within a prespecified quota design, with 100 patients per group receiving either platelet‐rich plasma plus lidocaine or lidocaine alone. Propensity score matching was performed to balance confounders. The primary outcome was the mean change from baseline in monthly headache days at 1 month post‐intervention. Secondary outcomes included headache frequency, pain intensity, headache duration, response rate, analgesic consumption, functional status, and safety.

**Results:**

After propensity score matching, 66 patients per group were analyzed. At one month, the platelet‐rich plasma plus lidocaine group showed a significantly greater reduction in monthly headache days compared with lidocaine alone (mean difference, 1.84 days; 95% confidence interval [CI], 0.68–2.99; *p* = 0.002). This advantage persisted at two and three months. The combination group also demonstrated superior improvements in pain intensity, headache duration, response rate, and analgesic reduction at all time points, along with better functional status at 1 and 2 months (all nominal P < 0.05). Baseline disability was a negative predictor of one‐month response. No significant differences in safety outcomes were observed.

**Conclusion:**

In this exploratory study, stellate ganglion block with platelet‐rich plasma plus lidocaine was associated with clinically meaningful improvements in headache outcomes and functional status compared with lidocaine alone in patients with chronic migraine, with a favorable safety profile. These findings warrant validation in a definitive randomized trial.

## 1 Introduction

Chronic migraine (CM), a disabling headache disorder affecting 1% to 2% of the global population, is defined as ≥15 headache days per month for at least 3 months (Gribbin et al. 2021; Headache Classification Committee of the International Headache Society (IHS) 2018). Its pathophysiology remains incompletely understood, with multiple factors contributing to the transformation from episodic migraine (EM) to CM (Aurora et al. 2007; Su and Yu 2018). Unlike EM, CM persists for months to years, severely impacting physical and mental health, interpersonal relationships, productivity loss and financial implications. Regrettably, at present, CM is often undertreated, and treatment outcomes are suboptimal (Kung et al. 2023 Feb).

Pharmacotherapy serves as the first line of CM treatment, including acute and preventive treatments. The approach to managing acute migraine attacks in CM patients typically starts with nonsteroidal anti‐inflammatory drugs (NSAIDs). If ineffective, triptans could be considered (Lipton et al. 2000). However, these acute medications have limited efficacy and intolerable side effects. And they are restricted to ≤2 days/week to avoid medication overuse headaches (MOH).5 A new class of drugs targeting the calcitonin gene‐related peptide (CGRP) pathway, including CGRP monoclonal antibodies and gepants (oral CGRP receptor antagonists), have been developed for the treatment of CM (Detke et al. 2018; Lipton et al. 2020). But these drugs are expensive and are usually reserved for cases where conventional drugs are ineffective, contraindicated, or cause unacceptable side effects (Lipton et al. 2000; Tepper et al. 2020). In clinical practice, beta‐blockers, antiseizure agents, calcium channel blockers, etc. are often used for CM preventive treatment (Dodick and Silberstein, 2007 Nov; Ford et al. 2024). Regrettably, it is difficult for patients to adhere to these medications. Therefore, for the substantial subgroup of patients with pharmacologically refractory or intolerant CM, exploring more effective treatment methods beyond pharmacotherapy has become a necessity.

Several percutaneous interventions for such refractory CM have been explored. A meta‐analysis involving 1139 patients found that injecting 100–195 units of Botulinum toxin A could reduce the monthly headache frequency by an average of 1.92 days compared to placebo (Giri et al. 2023 Apr). But it may increase the incidence of neck pain or muscle weakness and requires administration by trained healthcare professionals, increasing healthcare costs. Greater occipital nerve block has also shown clinical benefits, with a reported 34% reduction in monthly headache days (MHD) and 50% of cases reverting to EM after 1 month (Chowdhury et al. 2021). Nevertheless, its long‐term efficacy needs to be further confirmed. Neurostimulation techniques such as transcranial magnetic stimulation (TMS) and vagus nerve stimulation (VNS) are also applicable for CM (Hervias 2024). But their curative effects are uncertain, and their high costs impede widespread clinical adoption (Knotkova et al. 2021). Stellate ganglion block (SGB) with local anesthetic has been found beneficial for CM, as it inhibits sympathetic hyperactivity, induces vascular relaxation, enhances the delivery of trophic factors, and exerts anti‐inflammatory effects. A case report by Moon et al. (2020) showed that two CM patients who underwent bilateral SGB with 5 milliliters (mL) of 1.5% lidocaine seven times had a 4‐point decrease in their visual analogue scale (VAS) score after 4 months of treatment. Yu et al. (2023) also reported that CM patients who received an average of 2.97 SGB sessions with 5 mL of 1% lidocaine had effective rates of 90.7%, 82.5%, and 71.1% at 1, 2, and 3 months after the last SGB treatment, respectively. Due to the limited efficacy of a single SGB treatment, multiple treatments are often required, which restricts its practical application (Luo et al. 2022). It is of great significance to develop more effective SGB formulas to enhance the analgesic effect.

Platelet‐rich plasma (PRP), an autologous blood product with a platelet concentration 3 to 5 times higher than that in healthy whole blood (Knezevic et al. 2016), has emerged as an interventional treatment for neuropathic pain via modulating local inflammation and promoting axonal regeneration (Everts et al. 2020; Lai et al. 2022 Jul 1). Although CM pathophysiology is distinct, it involves peripheral and central sensitization in which neurogenic inflammation plays a critical role. PRP may counteract these pathological processes through its anti‐inflammatory, neurotrophic, and nerve‐repair properties. Supporting this rationale, Stone et al. (2024) demonstrated PRP alleviated post‐traumatic headaches within the greater occipital nerve distribution area. Based on the synergistic mechanisms of inflammation inhibition and neurotrophic support between SGB and PRP, we hypothesized their combination for SGB could yield enhanced effects in pain relief. However, this effect has not yet been reported. Given that a randomized controlled trial (RCT) was deemed premature, this prospective, exploratory observational study with propensity score‐matched analysis was conducted, guided by prioritizing patients' wishes, to evaluate whether a single SGB using PRP combined with lidocaine is more effective than a single SGB using lidocaine alone for refractory CM.

## 2 Methods

### 2.1 Study Design and Participants

This was a multi‐center PSM analysis of a prospective, exploratory cohort study (NCT06653218, registered on October 20, 2024) involving patients from the pain management departments of three hospitals in China: Capital Medical University Beijing Tiantan Hospital, Tongji Medical College Union Hospital, Huazhong University of Science and Technology, and Qinghai Provincial People's Hospital. This study was conducted from October, 2024, to September, 2025. The first patient was enrolled on October 25, 2024. Treatment allocation was primarily based on patient preference after detailed counseling. To ensure adequate and equal sample sizes for a robust comparative analysis between the two strategies, we employed a prospective quota sampling method. Specifically, we pre‐defined a recruitment target of 100 patients for each treatment arm: PRP + lidocaine group (receiving SGB with PRP + lidocaine) or lidocaine group (receiving SGB with lidocaine alone). Enrollment continued sequentially until each quota was filled. Thus, the final pre‐matching cohort comprised exactly 100 patients per group.

Patients who met the following criteria were enrolled: (1) 18–75 years; (2) body mass index 15–35 kg/m^2^; (3) diagnosed with CM according to the International Classification of Headache Disorders, third Edition (ICHD‐3); (4) refractory CM, defined as failure, intolerance, or contraindication to at least two classes of standard guideline‐based preventive therapies (Schulman et al. 2008) and additionally requiring a mean VAS score ≥4 during headache attacks at enrollment to ensure sufficient symptom burden; and (5) planned to receive SGB treatment for CM. Patients who met the following criteria were enrolled: (1) 18–75 years; (2) body mass index (BMI) 15–35 kg/m^2^; (3) diagnosed with CM in accordance with the International Classification of Headache Disorders, third Edition (ICHD‐3) criteria; (4) had a mean VAS score ≥4 after failure of, intolerance to, or contraindication for at least two classes of standard guideline‐based preventive therapies; and (5) planned to receive SGB treatment for CM.

Patients were excluded if they met the following criteria: (1) a history of prior SGB treatment; (2) comorbidity with other types of headaches; (3) platelet count <105 × 10^9^/L; (4) current use of anticoagulants or antiplatelet agents; presence of coagulation or bleeding disorders; (5) infection or mass near the puncture site; (6) history of other neurological disorders; (7) severe cardiopulmonary, hepatic, or renal dysfunction; (8) severe psychological disorders; (9) narcotic drug abuse; (10) neck anatomical structural changes due to radiotherapy or surgery; (11) allergy to study drugs; (12) pregnancy or lactation. The study was approved by the Ethics Committee of Beijing Tiantan Hospital (approval number: KY2023‐263‐03‐06). Its conduct and reporting adhered to the Strengthening the Reporting of Observational Studies in Epidemiology (STROBE) statement for cohort studies (Von Elm et al. 2007). All eligible patients provided written informed consent before screening.

**FIGURE 1:**
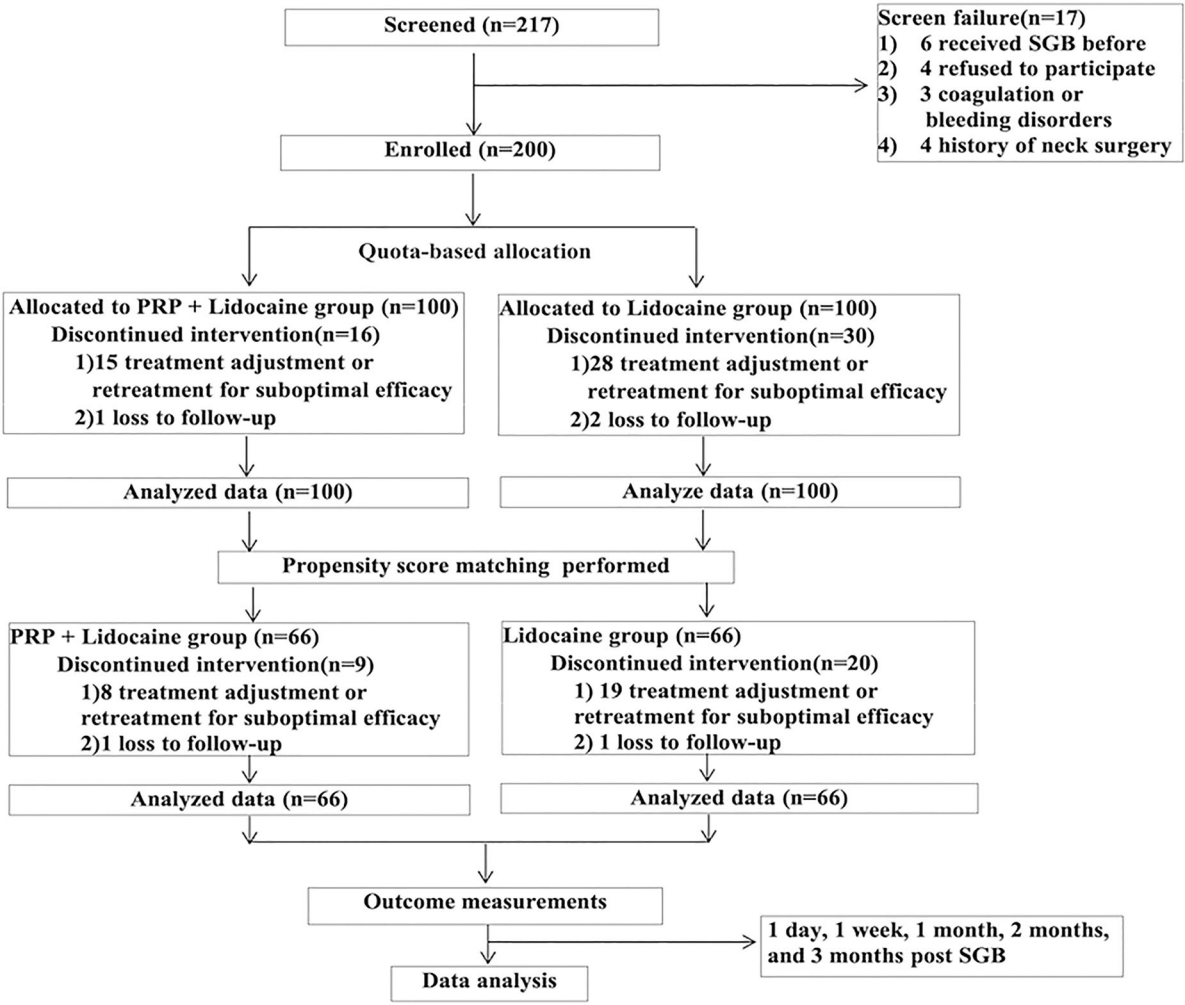
Study Flowchart. Flow diagram showing patient screening, quota‐based enrollment, treatment allocation, discontinuation, and propensity score matching (PSM) procedures. A total of 217 patients were screened, and 200 were enrolled and allocated to either the platelet‐rich plasma combined with lidocaine (PRP + lidocaine) group or the lidocaine‐only group. After PSM, 66 patients in each group were included in the primary analysis cohort. Abbreviations: PRP, platelet‐rich plasma; SGB, stellate ganglion block.

This observational study did not involve any intervention beyond routine clinical care. At each study center, a designated researcher who had received comprehensive training on the research protocol was responsible for recruiting CM patients based on the inclusion and exclusion criteria. The study flowchart, illustrating the quota‐based enrollment and subsequent matching process, was presented in Figure 1.

### 2.2 Intervention

**FIGURE 2:**
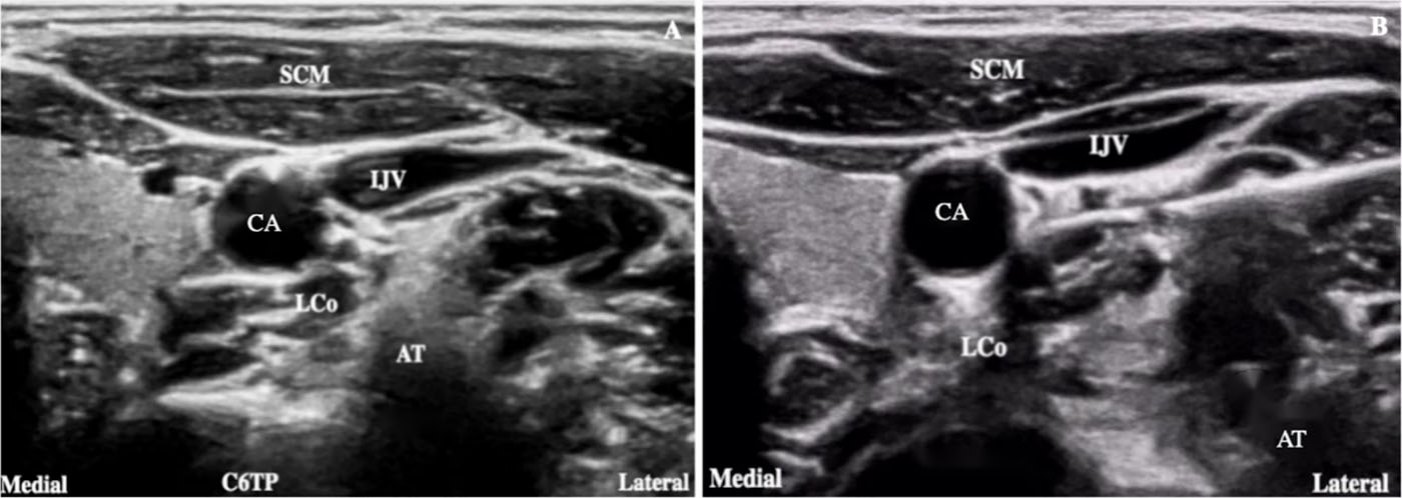
Ultrasound‐guided Stellate Ganglion Block Procedure. Short‐axis view of the cervical transverse process before injection, showing relevant anatomical landmarks including the common carotid artery (CA), internal jugular vein (IJV), anterior tubercle (AT), longus colli muscle (LCo), and the sixth cervical transverse process (C6TP). (B) Ultrasound image during the injection procedure, confirming correct needle placement and drug dispersion (arrows) on the surface of the longus colli muscle, with upward displacement of the common carotid artery indicating successful injection. Abbreviation: SCM, sternocleidomastoid muscle.

SGB procedure: All enrolled patients underwent ultrasound‐guided SGB performed by a designated, experienced pain physician at each center. Patients were positioned supine with neck extension. Heart rate, electrocardiogram, and pulse oximetry were monitored continuously. Blood pressure was measured every five minutes. Prior to the procedure, relevant anatomical landmarks, including the sixth cervical (C6) transverse process (C6TP), the anterior tubercle (AT) of C6TP, the thyroid gland, and the longus colli (LCo) muscle, were identified in the short axis view (Figure 2A). Following routine sterilization, a sterile 25‐gauge 38 mm sterile needle (Becton Dickinson Medical (S) Pte. Ltd.) was carefully inserted via an in‐plane approach to the superficial surface of the LCo muscle. Under continuous ultrasound guidance, 5 mL of the study drug was injected at a constant speed. Successful injection was confirmed by upward “floats” of the common carotid artery (Figure 2B). Post‐procedure, patients were monitored for at least 30 min to detect and rule out any SGB‐related complications.

PRP preparation: On the treatment day, autologous leukocyte‐poor PRP was prepared at 22–26 C using a cellular centrifugation system (WG‐YLJ‐II, WEGO, China). For patients with a unilateral headache, 25 mL of venous blood was drawn from the median cubital vein using a 22‐gauge, 1‐inch needle; for those with a bilateral headache, 50 mL was collected. The blood was immediately transferred into pre‐labeled sterile centrifuge tubes (marked with patient name and age) and gently mixed with acid citrate dextrose (ACD) at a ratio of 10:1.5 for anticoagulation (Liu et al. 2025). PRP preparation involved a two‐step centrifugation process. First, the blood was centrifuged at 1200 revolutions per minute (rpm) for 10 min to separate the plasma‐buffy coat from the lower red blood cell layer. Second, the upper plasma was transferred to a sterile, anticoagulant‐free tube and centrifuged at 1450 rpm for 15 min for further purification. After discarding the upper supernatant, approximately 2.5 mL (for unilateral headache) or 5 mL (for bilateral headache) of PRP was obtained. This PRP was vigorously shaken to ensure platelet activation before injection.

Treatment groups: All enrolled patients continued their prescribed preventive and acute medications according to standard treatment guidelines throughout the study period (Tassorelli et al. 2018; Hovaguimian and Roth 2022). Besides this, the treatment regimens for SGB were as follows: (1) For patients with unilateral headache: patients in the Lidocaine group received 5 mL of 1.0% lidocaine for ipsilateral SGB to treat CM. Patients in the PRP + Lidocaine group underwent ipsilateral SGB with a slow injection of 2.5 mL of PRP combined with 2.5 mL of 2.0% lidocaine (total 5 mL, final lidocaine concentration ∼1.0%); (2) For patients with bilateral headache, all patients underwent bilateral alternating SGBs, with a 30‐minute interval between the two sides (Moon et al. 2020). Patients in the Lidocaine group received 5 mL of 1.0% lidocaine at each side of stellate ganglion. Patients in the PRP + Lidocaine group received 2.5 mL of PRP combined with 2.5 mL of 2.0% lidocaine at each side of the stellate ganglion. During the 3‐month observation period, patients continued their previous analgesic medications, and dosages were adjusted as needed based on their clinical condition.

### 2.3 Data Collection and Follow‐Up

On the enrollment day, systematically trained researchers at each center conducted a structured interview to collect baseline data comprised: (1) demographics: age, sex, height, weight; (2) comorbidities: hypertension, diabetes mellitus, coronary heart disease and anxiety or depression status (Hospital Anxiety and Depression Scale (HADS) ≥ 11) (Beekman and Verhagen, 2018 Jul); (3) clinical features: aura presence, pain laterality (left/right/bilateral), disease duration, family history of CM, treatment history (prior anti‐CM therapy, including preventive medications, acute analgesics, and/or interventional procedures); (4) headache metrics recalled for the month before enrollment: monthly headache days (MHD), mean VAS score during headache attack, mean headache duration per episode; (5) HIT‐6 (Headache Impact Test version 6) score (Yang et al. 2011), Migraine Disability Assessment (MIDAS) score (Stewart et al. 2001), and Pittsburgh Sleep Quality Index (PSQI) score (Ashina et al. 2021 Jul 26). A headache attack was defined as an episode fulfilling the diagnostic criteria for migraine headache; administration of migraine‐specific acute medication; a headache episode recurring within 48 h after initial resolution; or a headache episode showing initial response to medication but relapsing within 48 h with total duration exceeding 48 h (Diener et al. 2020 Sep). On the SGB day, the pain physician documented vital signs (blood pressure, pulse, and respiratory rate), procedural details, and intraprocedural SGB‐related adverse events (AEs) in CRFs. After SGB, all patients were provided with a pain diary (PD) and instructed to prospectively record all subsequent headache metrics (time of headache onset, VAS score during attack, and headache duration per episode) and total daily analgesic consumption) one hour before bedtime every evening.

A designated, well‐trained outcome assessor at each study center conducted regular follow‐ups at 1 day, 1 week, 1 month, 2 months, and 3 months post‐procedure to collect PD data and administer follow‐up questionnaires. If a patient initiated any new interventional therapy for migraine or made a substantive change to their existing preventive medication regimen (e.g., addition, discontinuation, or dose escalation beyond the protocol) during the study period, this was recorded as a “treatment adjustment.” For such patients, follow‐up for efficacy outcomes was terminated at the time of adjustment. Efficacy analyses were based on the last available data collected prior to the treatment change.

Outcome assessors monitored and prompted PD completion and provided support for study‐related queries 24 h a day, 7 days a week. Due to the nature of the treatment assignment based on patient preference, blinding of patients and treating physicians was not feasible. However, to maintain blinding, outcome assessors were physically separated from the clinical team and were not present during the SGB procedure; all patient records and case report forms used only study‐specific identification numbers, with treatment group information stored separately in a password‐protected file accessible only to the study coordinator. Follow‐up visits were conducted in a separate area away from the treatment room, and patients were explicitly instructed at the beginning of each follow‐up visit not to disclose which treatment they had received; assessors were trained to interrupt any patient who inadvertently began to discuss their treatment allocation.

### 2.4 Study Outcomes

The primary outcome was the mean change from baseline in MHD at 1 month post‐SGB. Secondary outcomes included: (1) change in MHD: the mean changes from baseline in MHD at 2 and 3 months post‐SGB; (2) values of headache metrics at 1, 2, and 3 months after SGB: MHD (total headache days per 1‐month period), mean month VAS score (total of peak VAS scores per episode/number of episodes per 1‐month period), and mean headache duration per episode (total duration/number of episodes per 1‐month period); (3) pain relief at 1, 2, and 3 months after SGB: monthly response rate (≥50% reduction in headache days) and proportion of patients achieving >50% reduction in monthly analgesic consumption from baseline; (4) functional status at 1, 2, and 3 months after SGB: proportion of patients with HIT‐6 score ≥50, MIDAS score ≥11, and PSQI score > 7; (5) predictors: factors associated with the 1‐month response rate in the PRP + Lidocaine group; and (6) AEs (including procedure‐related and medication‐related AEs) at 1 day, 1 week, 1 month, 2 months, and 3 months of follow‐up.

### 2.5 Statistical Analysis

We used PSM to reduce the influence of potential confounding between the PRP + Lidocaine group and the Lidocaine group (Rosenbaum and Rubin 1983). The propensity score was calculated by using multivariate logistic regression models, which included age, gender, BMI, study center, disease duration, pain laterality, comorbidities, previous interventional treatment, baseline MHD, and baseline mean headache duration per episode (reported by the patients during the enrollment interview). We used 1:1 nearest‐neighbor matching without replacement (caliper width: 0.2 standard deviation of the logit propensity score) to balance baseline characteristics between groups, with standardized mean differences (SMDs) evaluated balance (Austin, 2009 Nov 10).

Sample size was estimated based on pilot study data and clinical experience. A total sample size of 200 was estimated to provide 90% power (two‐tailed α = 0.05) to detect a between‐group difference of 2.0 days in the reduction of MHD, assuming a standard deviation of 9.0 days for the change from baseline, based on pilot data and consistent with published real‐world studies reporting SDs of 8.83‐9.15 days for MHD change (Alsaadi et al. 2022 Jun 16). This estimate intentionally adopted a conservative SD to ensure adequate power under more variable conditions. Post‐hoc, the observed variability in the change score was lower than the assumed value used for sample size estimation (SD 4.4–5.4 vs 9.0), which suggests that the trial was sufficiently powered. After accounting for an anticipated 20% loss to follow‐up, this sample size was considered sufficient to allow for robust PSM and ensure adequate matched cohorts for the analysis.

All analyses were performed on both the full cohort and the PSM‐matched cohort using SPSS 30.0 (IBM Corp., Armonk, NY, USA). Missing data were handled using the last observation carried forward (LOCF) method. The PSM‐Matched Cohort (*n* = 132) was the primary population for efficacy conclusions. Comparisons between two groups of change from baseline in MHD were performed using analysis of covariance (ANCOVA), adjusting for baseline MHD as a covariate. Results are presented as adjusted mean difference with 95% confidence interval (CI) and *p*‐value. For outcomes measured at multiple time points, repeated‐measures ANOVA was used to examine time, group, and time‐by‐group interaction effects. Post‐hoc between‐group comparisons at individual time points were performed with independent t‐tests or Mann‐Whitney U tests, as appropriate. Continuous data are presented as mean ± standard deviation (SD) or median (interquartile range). Categorical data are presented as n (%) and compared using Chi‐square or Fisher's exact tests. A multivariable logistic regression analysis within the PSM‐matched PRP + Lidocaine group using backward stepwise selection (exit criterion P > 0.10) was performed on the PSM‐matched subset. Model fit was assessed using the Hosmer–Lemeshow test. Results are reported as odds ratios (OR) with 95% CI. All statistical tests were two‐tailed, with *p* < 0.05 considered statistically significant. To assess the sensitivity of the findings to differential attrition and LOCF imputation, two sensitivity analyses were performed in the PSM‐matched cohort. First, a complete‐case analysis included only patients who completed the full 3‐month follow‐up without treatment adjustment or loss to follow‐up; this fixed population comprised 57 patients in the PRP + Lidocaine group and 46 patients in the Lidocaine group, with MHD reduction and responder status evaluated separately at 1, 2, and 3 months. Second, a treatment‐failure‐equals‐non‐response (TFNR) analysis was applied to the binary responder outcome: patients who discontinued because of suboptimal efficacy were classified as non‐responders from the corresponding treatment‐failure assessment onward, whereas patients lost to follow‐up for reasons unrelated to efficacy retained their LOCF status; the denominator was fixed at 66 patients per group. TFNR was not applied to continuous MHD reduction because classification as non‐response does not define a unique continuous MHD value. For responder outcomes, risk ratios (RRs) were calculated as the responder proportion in the PRP + Lidocaine group divided by that in the Lidocaine group, with *p*‐values derived from the chi‐squared test. Given the exploratory nature of the numerous secondary endpoints, no formal adjustment for multiple comparisons was applied; P‐values for secondary outcomes should be interpreted with caution, focusing on effect sizes and clinical relevance.

## 3 Results

### 3.1 Baseline Characteristics

A total of 217 patients across three hospitals underwent SGB with PRP + lidocaine or lidocaine alone. After excluding 17 patients meeting predefined criteria, 200 CM patients were actually enrolled. Of these 200 patients, 46 had missing follow‐up data: 43 cases were attributed to treatment adjustment or retreatment for suboptimal efficacy (PRP + Lidocaine group: 2 patients at 1 month post‐SGB, 5 patients at 2 months post‐SGB, and 8 patients at 3 months post‐SGB; Lidocaine group: 6 patients at 1 month post‐SGB, 9 patients at 2 months post‐SGB, and 13 patients at 3 months post‐SGB). The remaining 3 cases were due to loss to follow‐up. They were all imputed using the LOCF method. Ultimately, all 200 patients were included in the final analysis. After PSM, 132 patients (66 matched pairs) constituted the primary analysis cohort. In this cohort, 29 patients missed follow‐up data, similarly managed by LOCF. 27 cases were due to suboptimal efficacy (PRP + Lidocaine group: 1 patient at 1 month post‐SGB, 2 patients at 2 months post‐SGB, and 5 patients at 3 months post‐SGB; Lidocaine group: 3 patients at 1 month post‐SGB, 5 patients at 2 months post‐SGB, and 11 patients at 3 months post‐SGB). Another two cases were due to loss to follow‐up.

The baseline data of the two groups before and after PSM are detailed in Table 1. Before matching, the PRP + Lidocaine group had a significantly longer disease duration, longer mean headache duration per episode, and higher rates of anxiety/depression and prior interventional therapy (all *p* < 0.01). After PSM, all baseline covariates were well‐balanced between the two groups, with all standardized mean differences (SMDs) below 0.1.

**TABLE 1:** Baseline data of the Two Groups (Before and after PSM).

	Before PSM				After PSM			
	PRP + Lidocaine Group	Lidocaine group	SMD	*p*‐value	PRP + Lidocaine Group	Lidocaine group	SMD	*p*‐value
Number, n	100	100	0.785		66	66	0.095	
Age (years, mean ± SD)	44.0 ± 12.7	43.2 ± 13.9	0.061	0.683	43.1 ± 12.9	43.0 ± 13.9	0.103	0.959
Female, n (%)	61 (61.0)	66 (66.0)	−0.102	0.463	43 (65.2)	38 (57.6)	0.124	0.371
BMI (kg/m^2^, mean ± SD)	23.2 ± 2.8	23.1 ± 2.8	0.045	0.748	23.5 ± 2.7	23.1 ± 2.9	0.063	0.438
Pain laterality, *n* (%)			−0.086	0.824			−0.167	0.813
Left	33 (33.0)	30 (30.0)			18 (27.3)	21 (31.8)		
Right	34 (34.0)	33 (33.0)			22 (33.3)	22 (33.3)		
Bilateral	33 (33.0)	37 (37.0)			26 (39.4)	23 (34.8)		
Aura symptoms, *n* (%)	24 (24.0)	33 (33.0)	−0.210	0.159	18 (27.3)	19 (28.8)	−0.035	0.846
Disease duration (months, mean ± SD)	14.7 ± 6.9	12.6 ± 4.4	0.312	0.009	13.3 ± 5.4	13.2 ± 3.9	0.053	0.927
Comorbidities, *n* (%)								
Hypertension	22 (22.0)	18 (18.0)	0.096	0.480	12 (18.2)	13 (16.7)	0.036	0.824
Diabetes	10 (10.0)	11 (11.0)	−0.033	0.818	6 (9.1)	7(10.6)	−0.050	0.770
Coronary heart disease	10 (10.0)	9 (9.0)	0.033	0.809	7 (10.6)	7 (10.6)	0.000	1.000
Anxiety or depression	39 (39.0)	22 (22.0)	0.347	0.009	20 (30.3)	18 (27.3)	0.093	0.701
Family history, *n* (%)	25 (25.0)	31 (31.0)	− 0.138	0.345	18 (27.3)	21 (31.8)	−0.035	0.567
Previous interventional therapy, *n* (%)	38 (38.0)	17 (17.0)	0.430	< 0.001	18 (27.3)	17 (25.8)	0.062	0.844
MHD, (days, mean ± SD)	20.8 ± 3.9	20.4 ± 4.8	0.123	0.440	20.6 ± 4.0	20.4 ± 4.7	0.109	0.814
Mean monthly VAS score, (score, mean ± SD)	6.7 ± 1.2	6.6 ± 1.3	0.126	0.388	6.7 ± 1.2	6.6 ± 1.3	0.055	0.779
Mean headache duration per episode (hours, mean ± SD)	17.1 ± 7.7	13.8 ± 4.6	0.422	< 0.001	14.8 ± 6.3	14.7 ± 4.7	0.025	0.923
Proportion of patients with HIT‐6 score ≥ 50, *n* (%)	61 (61.0)	59 (59.0)	0.041	0.885	34 (51.5)	43 (65.2)	−0.201	0.112
Proportion of patients with MIDAS score ≥ 11, *n* (%)	58(58.0)	50 (50.0)	0.161	0.256	33 (50.0)	34 (51.5)	−0.099	0.862
Proportion of patients with PSQI score>7, *n* (%)	67 (67.0)	64 (64.0)	0.063	0.655	42 (63.6)	44 (66.7)	−0.035	0.715

Abbreviations: BMI, Body Mass Index; MHD, monthly headache days; MIDAS, Migraine Disability Assessment Scale; PRP, platelet‐rich plasma; PSQI, Pittsburgh Sleep Quality Index; SD, standard deviation; SMD, standardized mean difference; VAS, Visual Analogue Scale;HIT‐6, Headache Impact Test‐6 items.

### 3.2 Efficacy Outcomes

**FIGURE 3:**
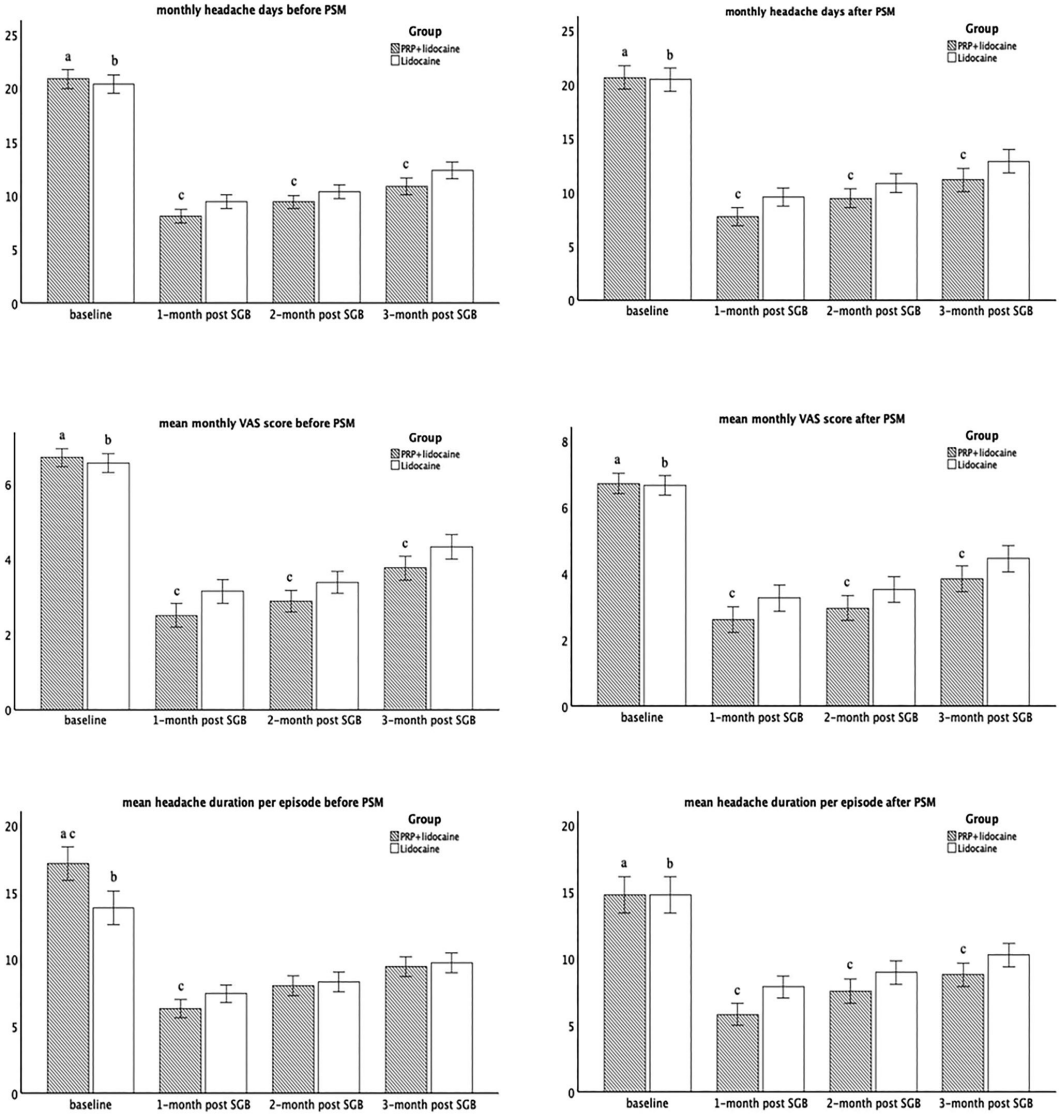
Changes in Headache Metrics Over Time (Before and After Propensity Score Matching). Longitudinal changes in monthly headache days from baseline to 3 months post‐intervention in the propensity score‑matched cohort. The PRP + Lidocaine group (blue line) demonstrated consistently greater reductions in monthly headache days compared with the Lidocaine‐only group (red line) at all follow‐up time points (1, 2, and 3 months). Error bars represent standard deviations. Between‐group differences were statistically significant at each time point (all nominal *p* < 0.05). Abbreviations: MHD, monthly headache days; PRP, platelet‐rich plasma; PSM, propensity score matching.

Headache metrics: Within the full cohort, the mean reduction in MHD at 1 month post‐SGB was 12.7 ± 4.8 days in the PRP + Lidocaine group compared to 10.9 ± 4.5 days in the Lidocaine group. After adjustment for baseline MHD, ANCOVA revealed a statistically significant between‐group difference of 1.41 days (95% CI: 0.55 to 2.26; P < 0.001). This advantage persisted at 2 and 3 months, with adjusted differences of 1.10 days (95% CI: 0.25‐1.94; *p* = 0.012) and 1.70 days (95% CI: 0.66‐2.73; *p* = 0.001), respectively. In the PSM‐matched cohort, the PRP + Lidocaine group showed a larger reduction in MHD at 1 month (12.9 ± 5.4 days vs. 10.9 ± 4.4 days), corresponding to an adjusted difference of 1.84 days (95% CI:0.68–2.99; *p* = 0.002). Significant between‐group differences were also observed at 2 and 3 months, with adjusted mean differences of 1.46 days (95% CI:0.29‐2.63; *p* = 0.015) and 1.80 days (95% CI: 0.39–3.22; *p* = 0.013), respectively. Similarly, the PRP + Lidocaine group demonstrated greater reductions in mean monthly VAS score and mean headache duration per episode at all follow‐ups (all nominal P < 0.05). Repeated‐measures ANOVA confirmed significant main effects of time and group for all headache metrics (all nominal *p* < 0.05), with post‐hoc tests showing consistently lower values in the PRP + Lidocaine group at each assessment (Figure 3).

Pain relief and functional status: In the PSM cohort, the PRP + Lidocaine group exhibited significantly higher responder rates and a greater proportion of patients achieving ≥50% reduction in analgesic consumption at 1, 2, and 3 months post‐SGB. Furthermore, the PRP + Lidocaine group showed more favorable outcomes in functional status measures (HIT‐6, MIDAS, PSQI) at the 1‐ and 2‐month assessments. Detailed comparisons are presented in Table 2.

**TABLE 2:** Comparison of pain relief and functional status between two group.

	Before PSM				After PSM			
	PRP + Lidocaine group	Lidocaine group	*p*‐value	RR (95%CI)	PRP + Lidocaine group	Lidocaine group	*p*‐value	RR (95%CI)
Response rate, n (%)								
1 month post‐treatment	86 (86.0)	63 (63.0)	<0.001	1.37 (1.16, 1.61)	55 (83.3)	42 (63.6)	0.010	1.31 (1.07, 1.61)
2 month post‐treatment	73 (73.0)	52 (52.0)	0.002	1.40 (1.14, 1.73)	47 (71.2)	34 (51.5)	0.020	1.38 (1.05, 1.82)
3 month post‐treatment	54 (54.0)	34 (34.0)	0.004	1.59 (1.16, 2.18)	36 (54.5)	24 (36.4)	0.036	1.50 (1.03, 2.19)
Proportion of patients with ≥ 50% reduction in monthly analgesic consumption								
1 month post‐treatment	66 (66.0)	41 (41.0)	< 0.001	1.61 (1.23, 2.10)	45 (68.2)	29 (43.9)	0.005	1.55 (1.14, 2.11)
2 month post‐treatment	56 (56.0)	35 (35.0)	0.003	1.60 (1.17, 2.19)	37 (56.1)	24 (36.4)	0.023	1.54 (1.06, 2.25)
3 month post‐treatment	44 (44.0)	28 (28.0)	0.018	1.57 (1.08, 2.29)	30 (45.5)	18 (27.3)	0.030	1.67 (1.05, 2.65)
Proportion of patients with HIT‐6 score ≥ 50, *n* (%)								
1 month post‐treatment	27 (27.0)	42 (42.0)	0.026	0.64 (0.43, 0.96)	14 (21.2)	29 (43.9)	0.005	0.48 (0.28, 0.83)
2 month post‐treatment	38 (38.0)	48 (48.0)	0.153	0.79 (0.57, 1.10)	19 (28.8)	31 (47.0)	0.031	0.61 (0.39, 0.96)
3 month post‐treatment	45 (45.0)	57 (57.0)	0.090	0.79 (0.60, 1.04)	30 (45.5)	36 (57.6)	0.164	0.83 (0.58, 1.19)
Proportion of patients with MIDAS score ≥ 11, *n* (%)								
1 month post‐treatment	21 (21.0)	38 (38.0)	0.008	0.55 (0.35, 0.87)	13 (19.7)	25 (37.9)	0.021	0.52 (0.29, 0.92)
2 month post‐treatment	26 (26.0)	42 (42.0)	0.017	0.62 (0.41, 0.93)	17 (25.8)	29 (43.9)	0.028	0.59 (0.36, 0.96)
3 month post‐treatment	39 (39.0)	47 (47.0)	0.253	0.83 (0.60, 1.15)	24 (36.4)	33 (50.0)	0.114	0.73 (0.49, 1.08)
Proportion of patients with PSQI score>7, *n* (%)								
1 month post‐treatment	26 (26.0)	43 (43.0)	0.011	0.60 (0.40, 0.91)	20 (30.3)	32 (48.5)	0.033	0.63 (0.40, 0.97)
2 month post‐treatment	31 (31.0)	46 (46.0)	0.029	0.67 (0.47, 0.97)	22 (33.3)	34 (51.5)	0.035	0.65 (0.43, 0.98)
3 month post‐treatment	38 (38.0)	52 (52.0)	0.047	0.73 (0.54, 1.00)	27 (40.9)	33 (50.0)	0.294	0.82 (0.56, 1.19)

*Note*: RR (Risk Ratio) was calculated as the proportion in the PRP + Lidocaine group divided by the proportion in the Lidocaine group. For response rate and analgesic reduction, RR > 1 indicates a higher proportion in the PRP + Lidocaine group; for HIT‐6, MIDAS, and PSQI scores, RR < 1 indicates a lower proportion in the PRP + Lidocaine group.

Abbreviations: CI, Confidence Intervals; HIT‐6, Headache Impact Test‐6 items; MIDAS, Migraine Disability Assessment Scale; PRP, platelet‐rich plasma; PSQI, Pittsburgh Sleep Quality Index; P‐values are nominal and unadjusted for multiple comparisons; RR, Risk Ratio.

Predictor analysis: Within the PSM‐matched PRP + Lidocaine group, multivariable logistic regression with backward stepwise selection identified a baseline MIDAS score ≥11 as a strong independent negative predictor of achieving a 1‐month response (OR = 0.041, 95% CI:0.004‐0.432, *p* = 0.008) (Table 3). In this exploratory analysis, the events‐per‐variable (EPV) ratio was 7.9, which falls below the recommended threshold of 10 for stable logistic regression models, and the Cl for baseline MIDAS ≥11 was extremely wide, indicating sparse‐data instability. Accordingly, these findings should be interpreted with caution.

**TABLE 3:** Predictors of 1‐month response in the PSM‐matched PRP + Lidocaine group.

Variable	B	SE	Wald	Sig.	Exp (B)	Lower 95% CI	Upper 95% CI
Baseline MIDAS score≥11(%)	−3.187	1.198	7.080	0.008	0.041	0.004	0.432
Constant	−18.981	8.076	5.524	0.019	0.000	—	—

Abbreviations: CI, confidence interval; MIDAS, Migraine Disability Assessment; PRP, platelet‐rich plasma; PSM, propensity‐score matching.

### 3.3 Sensitivity Analyses

To assess the robustness of the primary findings to differential attrition and LOCF imputation, we performed two pre‐specified sensitivity analyses for MHD reduction and responder rate in the PSM‐matched cohort (*n* = 132), including a complete‐case analysis and a TFNR analysis.

The complete‐case analysis yielded results consistent with the primary ITT‐LOCF analysis. In the complete‐case set, the adjusted mean difference for MHD reduction was numerically larger than that in the primary analysis, and the responder rate remained persistently higher in the PRP + lidocaine group.

In the TFNR analysis, the responder rate remained significantly higher in the PRP + lidocaine group across all follow‐up time points, although the magnitude of the effect estimates varied over time due to differential attrition between groups (Table 4).

**TABLE 4:** Sensitivity analyses for MHD reduction and responder rate in the PSM‐matched cohort.

Analysis set	Time–Point	Group	*n*	MHD Reduction (days, mean ± SD)	Adjusted mean difference (95% CI)	*p*‐value	Responder rate, n/N (%)	Risk ratio (95% CI)	*p*‐value
Complete‐case	1 month	PRP + Lidocaine group	57	12.6 ± 5.3	2.11 (0.73, 3.49)	0.003	47 (82.5)	1.58 (1.17, 2.14)	0.001
		Lidocaine group	46	10.4 ± 4.3	Reference		24 (52.2)	Reference	
	2 month	PRP + Lidocaine group	57	11.0 ± 4.7	1.46 (0.23, 2.68)	0.020	41 (71.9)	1.74 (1.19, 2.55)	0.002
		Lidocaine group	46	9.5 ± 4.5	Reference		19 (41.3)	Reference	
	3 month	PRP + Lidocaine group	57	9.4 ± 4.7	1.66 (0.14,3.18)	0.033	30 (52.6)	2.69 (1.42, 5.08)	< 0.001
		Lidocaine group	46	7.6 ± 4.9	Reference		9 (19.6)	Reference	
TFNR	1 month	PRP + Lidocaine group	66	NA	NA	NA	54 (81.8)	1.38 (1.10, 1.74)	0.004
		Lidocaine group	66	NA	NA	NA	39 (59.1)	Reference	
	2 month	PRP + Lidocaine group	66	NA	NA	NA	45 (68.2)	1.67 (1.19, 2.33)	0.002
		Lidocaine group	66	NA	NA	NA	27 (40.9)	Reference	
	3 month	PRP + Lidocaine group	66	NA	NA	NA	31 (47.0)	3.10 (1.66, 5.80)	< 0.001
		Lidocaine group	66	NA	NA	NA	10 (15.2)	Reference	

*Note*: In the TFNR analysis, patients who discontinued due to suboptimal efficacy retained their observed responder status at the discontinuation visit; all subsequent time points were imputed as non‑response. Patients who withdrew for other reasons (loss to follow‑up) retained their original last‑observation‑carried‑forward (LOCF) status. The denominator was fixed at 66 per group.

“NA” is presented for MHD reduction data in the TFNR analysis because no separate analysis was performed; these values are identical to those of the primary Intention‐to‐Treat (ITT) analysis.

Risk ratios were calculated with the lidocaine group as the reference. P values for responder rates were derived from the chi‑squared test.

Abbreviations: CI, confidence interval; MD, mean difference; MHD, monthly headache days; PRP, platelet‑rich plasma; PSM, propensity score matching; SD, standard deviation; TFNR, treatment failure = non‑response.

### 3.4 Adverse Events

The incidence of adverse events was similar between the two groups in both the full and matched cohorts (Table 5). No major complications (e.g., pneumothorax and intravascular injection) or PRP‐specific adverse events were reported. All recorded events were transient and resolved spontaneously without intervention.

**TABLE 5:** Adverse events by the Two Group before and after PSM.

Variable	Before PSM			After PSM		
	PRP + Lidocaine group	Lidocaine group	*p*‐value	PRP + Lidocaine Group	Lidocaine group	*p*‐value
Total adverse events, *n* (%)	23 (23.0)	29 (29.0)	0.333	16 (24.2)	18 (27.3)	0.691
Hoarseness, *n* (%)	3 (3.0)	4 (4.0)	0.700	1 (1.5)	2 (3.1)	0.559
Dysphagia, *n* (%)	1 (1.0)	2 (2.0)	0.561	0 (0.0)	1 (1.5)	1.000
Sore throat, *n* (%)	3 (3.0)	4 (4.0)	0.700	3 (4.6)	2 (3.1)	0.673
Cough, *n* (%)	2 (2.0)	3 (3.0)	0.651	2 (3.1)	1 (1.5)	0.559
Upper limb numbness, *n* (%)	1 (1.0)	3 (3.0)	0.312	1(1.5)	2 (3.1)	0.559
Dizziness, *n* (%)	1 (1.0)	2 (2.0)	0.561	1 1.5)	3 (3.1)	0.310
Puncture site pain, *n* (%)	12 (12.0)	11 (11.0)	0.825	8 (12.1)	7 (10.6)	0.784

Abbreviations: PRP, platelet‐rich plasma; PSM, propensity‐score matching.

## 4 Discussion

To our knowledge, this multicenter, prospective, PSM cohort study offers the first clinical evidence suggesting that augmenting a standard lidocaine SGB with autologous PRP may be associated with additional therapeutic benefits in patients with refractory CM.

In our study, both groups showed substantial reductions from baseline across all headache metrics, but the PRP + lidocaine combination consistently demonstrated greater improvements. At 1 month post‐intervention, the PRP‐augmented SGB group achieved a mean reduction in monthly headache days of 12.9 days (approximately 63% of baseline), compared with 10.9 days (53%) in the lidocaine‐only group, yielding an adjusted between‐group difference of 1.84 days (95% CI: 0.68–2.99). This advantage was most pronounced at 1 month and persisted, though with some fluctuation, through 2 and 3 months (adjusted differences: 1.46 and 1.80 days, respectively). A similar temporal pattern was observed for pain intensity and headache duration. This “peak effect within the first month followed by gradual decline” is consistent with the known transient nature of lidocaine‐induced sympathetic blockade and suggests that PRP may contribute additional biological effects beyond the transient blockade provided by lidocaine. However, a single PRP injection may not sustain the full therapeutic effect beyond three months. Repeated combination treatments may therefore be necessary to maintain long‐term efficacy, although the optimal treatment frequency and interval require further investigation.

In our study, the PRP‐augmented SGB group achieved a 1‐month response rate of 83.3%, compared with 63.6% in the lidocaine‐only group. By comparison, Yu et al. (2023) reported a 1‐month response rate of 90.7% and a 3‐month rate of 71.1% after an average of three conventional SGB sessions, while our corresponding 3‐month rates were 54.5% and 36.4%, respectively. These cross‐study comparisons are merely descriptive and should not be interpreted as suggesting equivalence between single PRP‐SGB and repeated conventional SGB. Differences in treatment frequency, patient populations (e.g., baseline characteristics and disease severity), study design (prospective vs. retrospective), analytical approaches (PSM vs. conventional regression), and outcome definitions preclude any direct inference of comparative efficacy. Head‐to‐head studies are warranted to determine whether PRP augmentation offers advantages over repeated conventional SGB.

Beyond headache frequency and intensity, PRP‐augmented SGB was associated with early improvements in functional status, including headache‐related quality of life, migraine‐related disability, and sleep quality at the 1‐ and 2‐month assessments. These functional benefits align with the contemporaneous reductions in pain, suggesting a cohesive therapeutic effect. However, these between‐group differences in functional status were not sustained at the 3‐month follow‐up. This time‐limited pattern mirrors the trajectory observed for headache metrics and may reflect that CM involves more complex psychological and social factors, requiring more comprehensive long‐term management strategies. Future studies with extended follow‐up periods could further explore the relationship between PRP combined therapy and sustained functional improvement, and determine whether repeated PRP‐augmented SGB can prolong or enhance these functional benefits.

Regarding the clinical significance of our findings, we acknowledge that the between‐group difference of 1.84 days in MHD reduction does not itself reach the conventional 2‐day MCID threshold adopted by some health technology assessment bodies (Pharmaceutical Benefits Advisory Committee (PBAC) July 2012). However, this does not diminish the clinical relevance of the combination therapy for several reasons. First, MCID is conventionally applied to within‐group change from baseline, and both groups substantially exceeded this threshold (12.9 days and 10.9 days), representing approximately 63% and 53% reductions from baseline. Second, the upper bound of the 95% CI (2.99) extends beyond the 2‐day threshold, meaning we cannot exclude the possibility of a clinically meaningful incremental effect. Third, the IHS‐recommended primary efficacy metric (responder rate) (International Headache Society (IHS) 2018) was significantly superior in the combination group (83.3% vs. 63.6%; RR = 1.31, 95% CI: 1.07–1.61), indicating that a substantially larger proportion of individual patients achieved a clinically meaningful response. Fourth, these primary endpoint findings were corroborated by consistent improvements in secondary outcomes, including pain intensity, headache duration, analgesic consumption, and functional status (HIT‐6, MIDAS, PSQI). Therefore, the clinical relevance of the combination therapy is supported by improvements across multiple clinically meaningful domains rather than by the primary endpoint alone.

The observed benefits may be attributed to the synergistic effects of PRP and lidocaine. Lidocaine provides immediate sympathetic blockade and anti‐inflammatory effects (Lin et al. 2020), while PRP, through its concentrated release of growth factors (e.g., platelet‐derived growth factor [PDGF], transforming growth factor‐beta [TGF‐β], and vascular endothelial growth factor [VEGF]) and anti‐inflammatory cytokines (e.g., interleukin‐1 receptor antagonist [IL‐1Ra]), may modulate the “neurovascular inflammation,” provide nutritional support, therefore producing a multi‐target intervention effect; and contribute to more sustained pain relief (Bohren et al. 2022 Jan). Of course, it should be noted, in vitro evidence also suggests lidocaine may attenuate certain PRP‐mediated cellular benefits, indicating that the therapeutic effects might be partially limited by the anesthetic choice. Future research should therefore explore alternative anesthetic agents to determine whether they can better preserve or even enhance the therapeutic benefits of PRP, thereby optimizing the combined treatment strategy.

We also performed an exploratory analysis to examine whether baseline characteristics were associated with treatment response. Within the PRP + lidocaine group, baseline MIDAS ≥11 showed a statistical association with lower odds of achieving a 1‐month response. The diminished efficacy observed in these patients may be attributable to more entrenched pain pathophysiology, such as central sensitization and a higher burden of psychosocial comorbidities (Dodick, 2018 Mar 31; Buse and Lipton 2013). These factors may contribute to a more complex and refractory condition, in which a single peripheral intervention like PRP may be insufficient to reverse established functional impairment (Lipton et al. 2000; Lantéri‐Minet et al. 2011). This observation aligns with the broader principle in chronic pain management that earlier intervention, undertaken before the establishment of severe functional impairment, may be associated with more favorable outcomes. However, this finding should be considered preliminary and hypothesis‐generating for two reasons. First, the analysis was based on a small sample size (66 patients in the PRP + lidocaine group), and the extremely wide confidence interval (OR = 0.041, 95% CI: 0.004–0.432) indicates sparse‐data instability and possible overfitting. Second, MOH was not assessed at baseline, and we cannot rule out residual confounding by this important factor, particularly its potential influence on MIDAS scores and other functional outcomes (Togha et al. 2020). If confirmed in future studies with larger sample sizes and detailed medication histories, the MIDAS score could serve as a useful tool for patient selection in clinical research.

Reports indicate that the most common symptoms of SGB are hoarseness and dizziness. Short‐term and persistent coughs have also been observed following SGB, which are thought to be associated with recurrent laryngeal nerve palsy (Atici and Akoz, 2010 May‐Jun). Other rare complications include accidental intravascular injection, intraoperative bleeding, and hematoma formation (Kirkpatrick et al. 2023 Feb 19). In our study, only minor complications were documented in both groups, suggesting the short‐term (up to 3 months) safety of SGB. Notably, image guidance and rigorous operator training can further reduce the incidence of complications. Moreover, the addition of PRP did not increase the occurrence of SGB‐associated complications, thereby validating the strong safety profile of the combined application.

## 5 Limitations

There are several limitations of this study. First, the study was non‐randomized and open‐label, with both patients and treating physicians aware of treatment assignment. Although we employed propensity score matching to balance measured covariates and blinded outcome assessors and statisticians to group allocation, unmeasured confounding and expectation bias cannot be fully excluded. This concern is particularly salient for patient‐reported outcomes, including the primary endpoint of monthly headache days and all secondary patient‐reported measures (VAS, headache duration, analgesic use, HIT‐6, MIDAS, and PSQI). Moreover, we did not formally verify the success of assessor blinding (e.g., by asking assessors to guess treatment allocation), although no unblinding events were reported. Second, baseline headache metrics were established through patient recall, introducing potential recall bias, and MOH was not systematically assessed, precluding adjustment for this well‐established confounder that may influence both treatment response and functional outcomes such as MIDAS scores. Third, differential attrition was greater in the lidocaine group (19 vs. 8 after matching). Although the complete‐case and TFNR sensitivity analyses showed a consistent direction of effect favoring the PRP + Lidocaine group, the magnitude of the relative effect varied with the handling of differential attrition. We therefore neutrally refer to the TFNR analysis as a sensitivity analysis, rather than a definitive conservative estimate. Consequently, some uncertainty remains, and the primary ITT‐LOCF analysis should be considered the preferred estimate. Fourth, the analysis of secondary outcomes was exploratory, and nominal P‐values are reported without adjustment for multiple comparisons, increasing the risk of Type I error. These findings should therefore be interpreted with caution. Fifth, the predictor analysis examining baseline MIDAS as a response modifier was underpowered (EPV = 7.9, with extremely wide CIs) and requires validation in larger prospective studies. Sixth, we did not verify platelet concentration or assess leukocyte content and activation status of the prepared PRP, limiting our ability to correlate clinical effects with specific platelet doses and weakening the biological rationale. Variability in centrifugation protocols across centers may further influence outcomes. Routine quality control metrics should be incorporated in future research to improve reproducibility. Seventh, the follow‐up duration was limited to 3 months, and we did not conduct a cost‐effectiveness analysis. Extended follow‐up and health economic evaluations are needed in future studies to determine whether the benefits of PRP justify its added cost.

## 6 Conclusion

In summary, this exploratory study suggests that for patients with refractory CM, a single SGB with PRP and lidocaine is associated with higher responder rates and consistent functional improvements compared with lidocaine alone over a 3‐month period. While the absolute between‐group difference in MHD was modest, the combination of superior responder rates, reduction in analgesic use, and improvements in quality‐of‐life measures supports the clinical relevance of this approach. These findings provide a strong rationale for further investigation in a definitive randomized controlled trial with longer follow‐up and standardized PRP preparation protocols.

## Author Contributions

**Lu Liu**: study concept and design, acquisition of data, analysis and interpretation, drafting the manuscript. **Baolin Duan**: acquisition of data and critical revision of the manuscript for important intellectual content. **Tao Ma**: acquisition of data and critical revision of the manuscript for important intellectual content. **Long Li**: acquisition of data and critical revision of the manuscript for important intellectual content. **Dong Yang**: study concept and design, acquisition of data and critical revision of the manuscript for important intellectual content. **Fang Luo**: study concept and design, critical revision of the manuscript for important intellectual content, supervision. All authors reviewed and approved the final version of the manuscript to be published.

## Funding

This work was supported by the National Key Research and Development Program of China (2022YFC3602200, 2022YFC3602201, 2022YFC3602202, 2022YFC3602203, and 2022YFC3602205) and the Health Improvement and Research (No. 2020‐1‐4061). The funders had no role in the study design, data collection, data analysis, data interpretation, or writing of the manuscript.

## Conflicts of Interest

The authors declare no conflicts of interest.

## Data Availability

The data that support the findings of this study are available on request from the corresponding author. The data are not publicly available due to privacy or ethical restrictions.
